# Fecal amino acid and short-chain fatty acid profiles in children with migraine: a targeted metabolomics study

**DOI:** 10.3389/fmicb.2026.1788386

**Published:** 2026-05-08

**Authors:** Ning Tian, Fan Yang, Yuan Zhao, Mei Jin, Feng Sun, Min Liu, Yufei Lian, Suzhen Sun, Kang Liu

**Affiliations:** 1 Neurology Department, Hebei Children's Hospital, Shijiazhuang, China; 2 International Liaison Office, Hebei Children's Hospital, Shijiazhuang, China; 3 The Key Laboratory of Pediatric Epilepsy and Neurological Disorders of Hebei Province, Shijiazhuang, China; 4 Department of Epidemiology and Biostatistics, School of Public Health, Peking University, Beijing, China; 5 Key Laboratory of Epidemiology of Major Diseases (Peking University), Ministry of Education, Beijing, China; 6 College of Life Sciences, Hebei Normal University, Shijiazhuang, China; 7 Pharmacy Department, Hebei General Hospital, Shijiazhuang, China

**Keywords:** children with migraine, intestinal microecology, phenylalanine, probiotics, short-chain fatty acids, tryptophan

## Abstract

**Background:**

Research has primarily focused on the gut microbiota of adult migraine patients; however, investigations into specific metabolic alterations in pediatric migraine remain limited, and comprehensive targeted metabolomics characterization in this population is still lacking. This exploratory cross-sectional study aims to identify specific metabolic signatures associated with pediatric migraine using targeted metabolomics.

**Methods:**

We enrolled 30 children with migraine and 30 healthy controls (with no history of headache) aged 5–14 years from Hebei Province, China, and collected 60 fresh fecal samples. Using targeted metabolomics, we profiled amino acids, their derivatives, and short-chain fatty acids (SCFAs) to compare fecal metabolite profiles between groups. Differentially altered metabolites were further analyzed through KEGG pathway enrichment and receiver operating characteristic (ROC) curve analysis.

**Results:**

Compared with healthy controls, eight amino acid metabolites—including 2,6-diaminopimelic acid, L-valine, L-leucine, and L-phenylalanine—were significantly elevated in children with migraine (*P* < 0.05). These differential metabolites were enriched in pathways related to the biosynthesis and degradation of valine, leucine, and isoleucine; the biosynthesis of phenylalanine, tyrosine, and tryptophan; and arginine biosynthesis. SCFA-related differences were also observed between groups; however, most individual SCFA comparisons did not reach statistical significance and should therefore be interpreted cautiously. Some of these differential metabolites were also mapped to pathways related to protein digestion and absorption. ROC curve analysis showed that tryptamine (AUC = 0.70, 95% CI: 0.56–0.84) and 2,6-diaminopimelic acid (AUC = 0.73, 95% CI: 0.60–0.87) had modest discriminative performance in distinguishing children with migraine from healthy controls.

**Conclusion:**

Children with migraine exhibit distinct metabolic signatures, particularly involving amino acid-related metabolites. The abnormal elevation of key amino acids, such as phenylalanine and tryptophan, was associated with pediatric migraine. SCFA-related differences were also observed. Furthermore, metabolites such as tryptamine and 2,6-diaminopimelic acid may represent candidate metabolites with preliminary discriminatory value for pediatric migraine, warranting further investigation in larger validation cohorts.

## 1 Introduction

Migraine is a common neurovascular disorder characterized by recurrent episodes, often accompanied by nausea, vomiting, and photophobia or phonophobia (Steiner et al., 2019). The etiology of pediatric migraine is recognized as multifactorial, involving an intricate interplay between genetic susceptibility (Hautakangas et al., 2022), environmental triggers (Eigenbrodt et al., 2021), and psychological stressors (Lim et al., 2022). According to 2021 data, approximately 281 million people worldwide suffer from migraine or tension-type headaches (GBD 2021 Diseases and Injuries Collaborators, 2024). The global prevalence of migraine is steadily increasing, particularly among younger populations (Steiner et al., 2019). In children, the prevalence of migraine is approximately 11% (Onofri et al., 2023), with the highest growth rate observed in those aged 10–14 years (Yang and Cao, 2023). Furthermore, the most recent Global Burden of Disease data indicate that migraine and associated neurological conditions significantly contribute to disability-adjusted life years among children and adolescents aged 5–19 years (GBD 2021 Nervous System Disorders Collaborators, 2021). This disease burden generally increases with age (GBD 2021 Diseases and Injuries Collaborators, 2024).

The brain development (Demeter et al., 2025), cognitive characteristics (Ismail, 2020), and psychophysiological profiles (Eccleston et al., 2020) of children differ substantially from those of adults. Consequently, pediatric migraine differs from adult migraine in several clinical features: it more commonly presents with bilateral headaches (Youssef and Mack, 2020), and the duration of headache episodes is typically shorter (Steiner et al., 2019). Moreover, during attacks, children with migraine often experience limitations in daily functioning (Kernick et al., 2009; Pann et al., 2024), reduced academic performance (Canfora et al., 2023; Genc et al., 2021), and even social difficulties (GBD 2021 Diseases and Injuries Collaborators, 2024). Although the International Headache Society has updated the diagnostic criteria for migraine, clinical management still relies primarily on acute analgesics and preventive medications (Patniyot and Qubty, 2020), which are associated with poor treatment adherence, frequent side effects, and considerable interindividual variability in efficacy (ÖzdemIr KaÇer and AteS, 2023). Treatment options for pediatric patients are even more limited. Therefore, exploring non-pharmacological interventions and improving our understanding of disease-associated biological changes hold significant clinical value.

The gastrointestinal tract is closely associated with neuroendocrine and metabolic signaling processes, and serves as a site for the production and regulation of various neurotransmitters (Qu et al., 2024), including serotonin and γ-aminobutyric acid (LaGreca et al., 2022). Children with migraine frequently experience recurrent gastrointestinal symptoms, including abdominal pain, during headache attacks (Arzani et al., 2020; Hindiyeh and Aurora, 2015), indicating that gut-related symptoms may accompany pediatric migraine. The development and maturation of the pediatric intestinal environment and its metabolic profile follow a unique trajectory, remaining in a dynamic phase throughout the first decade of life (Vatanen et al., 2022). This developmental context suggests that microbial and metabolic features in children may differ from those observed in adults; therefore, findings from adult migraine studies may not be directly generalizable to pediatric populations. In addition, Mendelian randomization analysis has suggested potential associations between specific intestinal constituents and the risk of migraine with and without aura (He et al., 2023). Previous studies have suggested that intestinal processes may influence systemic neurotransmitter metabolism (Zhu et al., 2020).

Probiotics are increasingly investigated as a means of modulating the gut metabolic environment. Studies in adults have suggested that synbiotic supplementation can influence these profiles and has been explored as an adjunct therapy for women with migraine, improving headache features and inflammatory markers while reducing disease burden (Ghavami et al., 2021). Recent pediatric gut studies further suggest that host phenotypes may be influenced not only by the intestinal environment itself (Roswall et al., 2021), but also by microbially derived metabolites and their interactions with host immune (Al Nabhani and Eberl, 2020; Henrick et al., 2021), neural (Kelsey et al., 2020), and metabolic pathways (Briend et al., 2022). In this context, metabolite-centered approaches may complement microbial profiling by providing functional readouts that reflect localized biological activity. Therefore, investigating fecal metabolites in pediatric migraine may provide complementary information on disease-associated metabolic patterns, serving as an observational baseline even in the absence of direct metagenomic data.

Among metabolite-centered approaches, targeted metabolomics offers high reproducibility and quantitative accuracy for the assessment of specific biochemical pathways (Cai and Zhu, 2019). Although prior studies have implicated altered amino acid metabolism (Domitrz et al., 2015) and SCFA-related differences (Zhu et al., 2020) in neurological disorders, these investigations predominantly focused on adult cohorts or systemic serum profiling. Compared with systemic blood, fecal targeted metabolomics may provide a more proximal assessment of intestinal metabolite profiles. Therefore, this study aimed to characterize fecal metabolic features in children with migraine through the targeted profiling of amino acids, their derivatives, and SCFAs. While these preliminary findings require validation in larger cohorts, they may help characterize fecal metabolite-level differences associated with pediatric migraine and inform future integrative studies combining metabolomics with microbiome and clinical data.

## 2 Methods

### 2.1 Participants

In this study, fecal samples were prospectively collected from 30 children with migraine and 30 healthy children between May 2024 and January 2025. All participants were recruited from inland regions of China and aged 5–14 years. They were assigned to either the migraine group (*n* = 30) or the healthy control group (*n* = 30). The diagnosis of pediatric migraine was established according to the internationally recognized ICHD-3 diagnostic criteria. The study protocol was approved by the Ethics Committee of Hebei Children's Hospital (Approval No.: 2024132), and written informed consent was obtained from all legal guardians.

#### 2.1.1 Inclusion and exclusion criteria

The inclusion criteria for the migraine group were as follows: (1) diagnosis of migraine according to the ICHD-3 criteria (Ghavami et al., 2021); (2) Han ethnicity, age 5–14 years, and headache duration exceeding 1 month; (3) treatment-naïve status at the time of enrollment, with no use of antibiotics, analgesics, sedatives, or immunomodulatory drugs within 2 weeks prior to baseline specimen collection; (4) not being in the menstrual period (for female participants); and (5) willingness of the child's guardian to participate, with full understanding of the study procedures and provision of signed informed consent.

Exclusion criteria for the migraine group were as follows: (1) presence of other primary or secondary headache disorders; (2) recent use of antibiotics, hormones, immunoglobulins, or other immunomodulatory therapies within 2 weeks prior to specimen collection; (3) diagnosis of gastrointestinal disorders such as inflammatory bowel disease or infectious diarrhea, or other significant systemic comorbidities; and (4) history of major organ failure.

Inclusion criteria for the healthy control group were: (1) age 5 and 14 years; (2) absence of chronic or acute medical conditions, no personal or family history of primary headache disorders, with no use of medications or dietary supplements within 2 weeks prior to specimen collection; and (3) provision of informed, written consent by the child's legal guardian.

### 2.2 Variables and data sources

#### 2.2.1 Sample collection

Fresh fecal samples were collected from participants at enrollment (baseline) using sterile spoons, ensuring no contact with air or environmental surfaces. Immediately after collection, each sample was placed into a sterile tube and stored at −80 °C. Fecal samples from healthy children were collected using the same standardized procedure.

#### 2.2.2 Clinical data collection

Baseline clinical characteristics—including age, sex, height, and prior medication use—were collected through structured medical history interviews and review of medical records. The severity of migraine was assessed using validated patient-reported outcome measures, including the Visual Analog Scale (VAS), the Pediatric Migraine Disability Assessment Score (Ped MIDAS), and the Headache Impact Test-6 (HIT-6).

### 2.3 Targeted metabolomics analysis

In this study, a precisely weighed quantity of each sample was used. Under low-temperature conditions, an extraction solvent was added to enable metabolite extraction, and calibration standard solutions of known concentrations were prepared. Both the standard solutions and test samples were then analyzed under identical analytical conditions using liquid chromatography–mass spectrometry (LC–MS). The concentration of each target metabolite in the sample was determined by interpolation from the corresponding calibration curve, allowing quantification of its actual content. Detailed experimental procedures are provided in the Supplementary Material.

### 2.4 Statistical analysis

In the AB Sciex quantitative software OS, default parameters were used for automatic identification and integration of each ion peak, followed by manual curation. A linear regression calibration curve was generated with the mass spectrometry peak area of the analyte as the ordinate and its concentration as the abscissa. Sample concentrations were determined by interpolating the analyte's peak area into the corresponding linear equation. Inter-group statistical comparisons were performed using *R* language (version 3.3.1). Differential analysis was conducted using the two-tailed Wilcoxon rank-sum test with False Discovery Rate (FDR) correction. KEGG pathway enrichment analysis was carried out using Python (version 2.7.10), and the significance of enriched pathways was validated using the FDR method. To evaluate the overall metabolic data distribution and quality, unsupervised Principal Component Analysis (PCA) with Pareto scaling was performed. Accordingly, differential metabolites with *P* < 0.05 were selected to minimize false-positive findings and enhance the robustness of the results. Additionally, the diagnostic performance of candidate biomarkers was evaluated *via* Receiver Operating Characteristic (ROC) curve analysis, with the Area Under the Curve (AUC) and 95% confidence intervals (CI) calculated to assess model stability. Spearman correlation analysis was used.

## 3 Results

### 3.1 Baseline characteristics of children with migraine and healthy controls

A total of 60 fresh stool samples were collected and analyzed, including 30 from children with migraine and 30 from healthy children. The migraine group consisted of 18 females (60.0%) and 12 males (40.0%) with a mean age of 10.47 ± 2.53 years, while the healthy control group included 14 females (46.7%) and 16 males (53.3%) with a mean age of 9.33 ± 2.12 years. No significant differences in sex (*P* = 0.305) or age (*P* = 0.073) were observed between the two groups. All participants were recruited from the same province (Hebei Province, China) and shared similar dietary habits, lifestyles, and living environments, thereby substantially minimizing the potential confounding effects of non-migraine-related factors.

### 3.2 Principal component analysis of fecal metabolites

**Figure 1:**
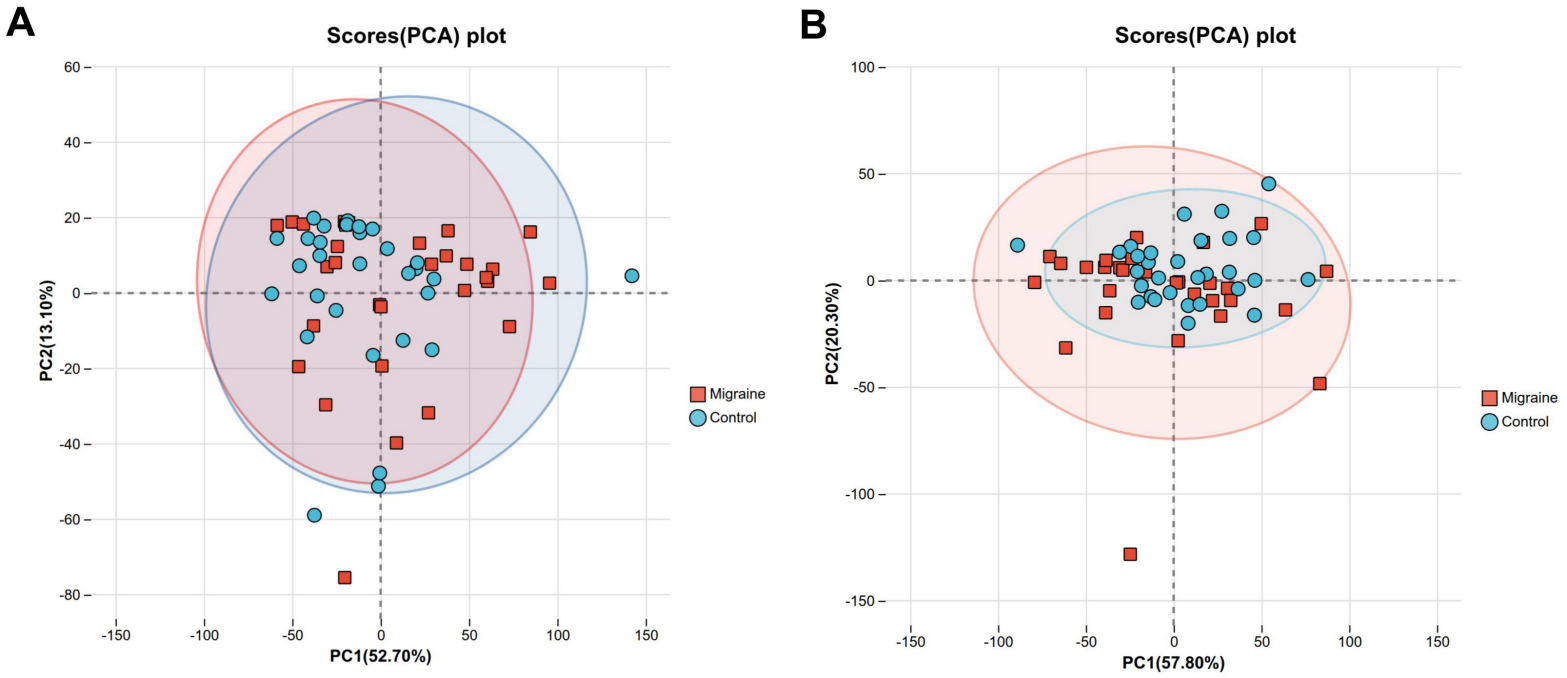
PCA score plots. *Note:* **(A)** PCA score plot of the amino acid metabolome (R^2^X = 0.658). **(B)** PCA SCFAs metabolome (R^2^X = 0.781). Each point represents an individual sample, with red and blue symbols indicating the migraine and healthy control groups, respectively. The shaded ellipses represent the 95% confidence intervals.

PCA of the targeted amino acid metabolome showed that PC1 and PC2 together explained 65.8% of the total variance. Similarly, PCA of the SCFAs dataset showed that the first two principal components accounted for 78.1% of the cumulative variance, supporting the overall stability of the analytical data (Figure 1).

### 3.3 Metabolite analysis of amino acids and their derivatives

**Figure 2:**
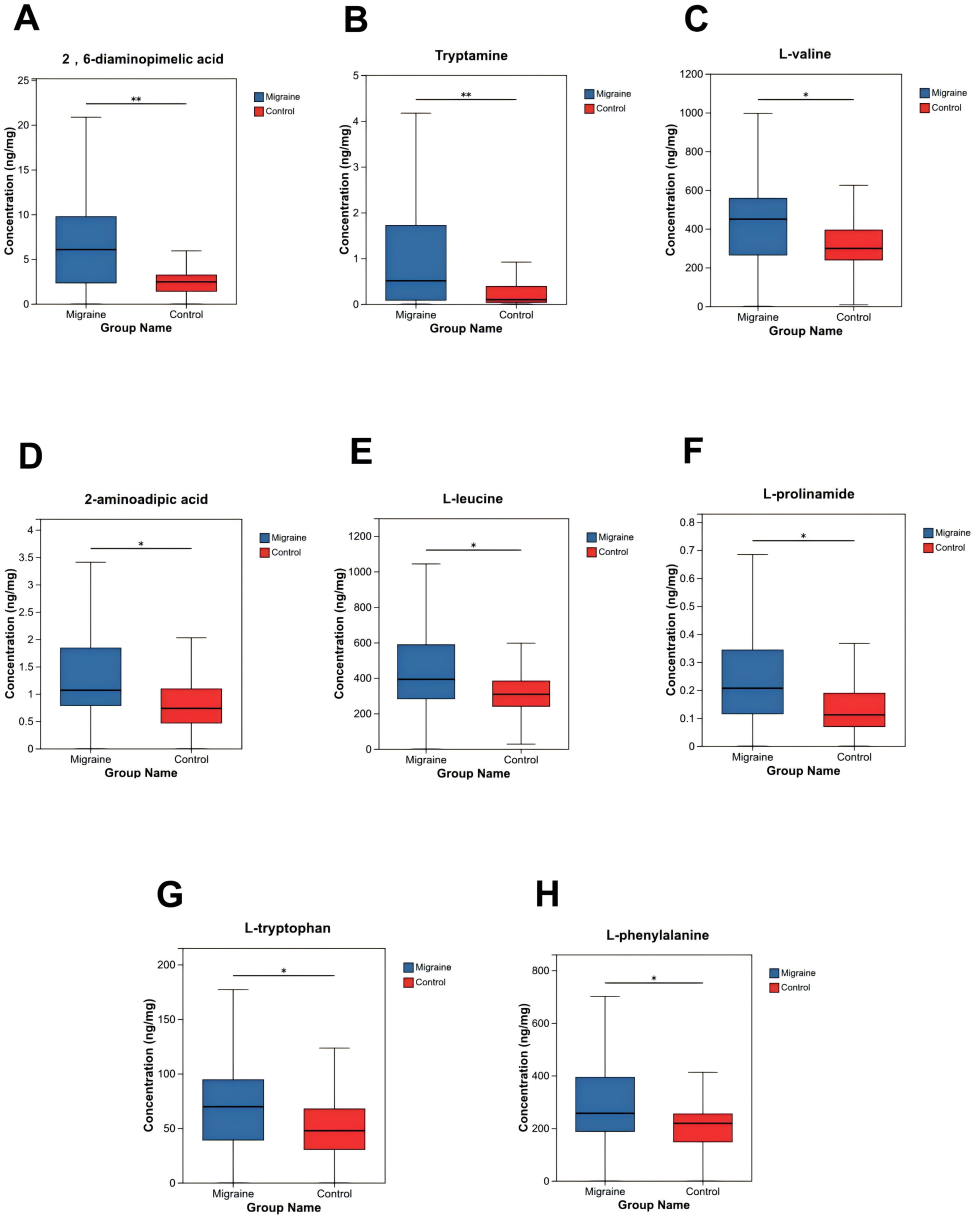
**(A)** 2,6-diaminopimelic acid; **(B)** Tryptamine; **(C)** L-valine; **(D)** 2-aminoadipic acid; **(E)** L-leucine; **(F)** L-prolinamide; **(G)** L-tryptophan; and **(H)** L-phenylalanine. *Note:* The abscissa indicates the two comparison groups, and the ordinate shows the mass spectrometry intensity (post-preprocessing). The data are presented as boxplots, where the central horizontal line represents the median relative abundance of the metabolite. The upper and lower edges of the box indicate the upper quartile (Q3) and lower quartile (Q1), respectively, with the height of the box reflecting the data's interquartile range (IQR) and fluctuation. The whiskers represent the maximum and minimum values within 1.5 times the IQR. Significance is denoted as follows: *0.01 < *P* < 0.05, **0.001 < *P* < 0.01, ***P < 0.001.

Targeted metabolomic analysis of the 60 samples identified a total of 66 amino acids and related metabolites. Differential metabolites were screened based on *P* value and fold change, revealing eight amino acids that differed significantly between groups (Wilcoxon rank-sum test, two-tailed, *P* < 0.05). Among these, key metabolites-−2,6-diaminopimelic acid, L-valine, L-leucine, and L-phenylalanine showed significantly higher levels in the migraine group compared to the control group (Table 1, Figure 2).

**Table 1:** Differential amino acid metabolites between the migraine and control groups.

Metabolite	*P*_ value	Regulation
2,6-diaminopimelic acid	0.002	UP
Tryptamine	0.008	UP
L-valine	0.013	UP
2-aminoadipic acid	0.020	UP
L-leucine	0.022	UP
L-prolinamide	0.022	UP
L-tryptophan	0.031	UP
L-phenylalanine	0.036	UP

**Figure 3:**
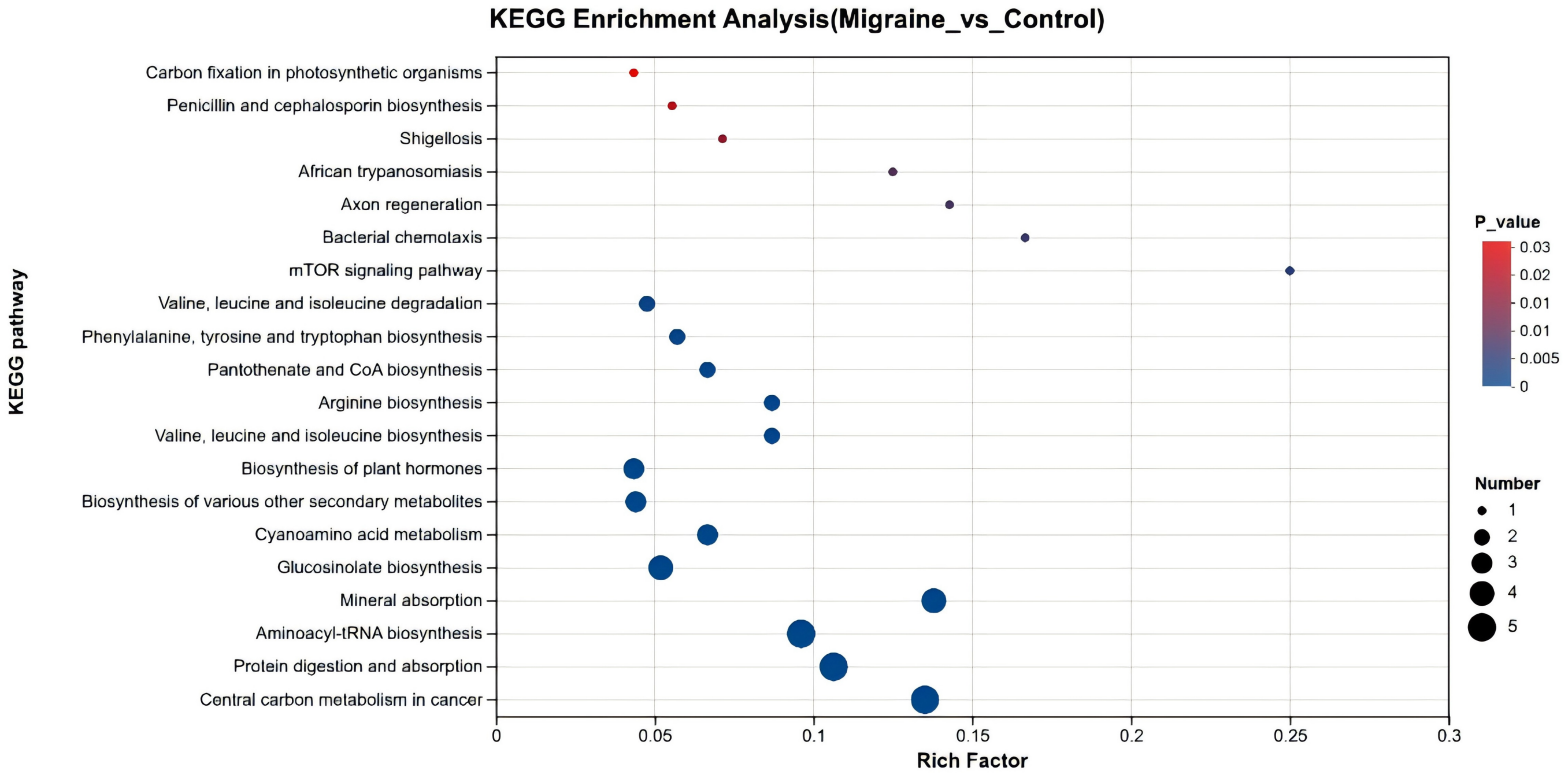
Differential amino acid KEGG enrichment analysis bubble diagram. *Note:* The abscissa represents the enrichment ratio, calculated as num_ in_ study / num_ in_ pop. The ordinate lists the KEGG pathways. In the figure, bubble size corresponds to the number of compounds enriched in each pathway, and bubble color indicates the significance level (–log10(*P*-value)) of the enrichment.

Based on the eight differentially abundant metabolites identified, these metabolites were subjected to KEGG pathway enrichment analysis. The analysis revealed that these key metabolic alterations were enriched in multiple KEGG pathways: (1) biosynthesis and degradation of valine, leucine, and isoleucine within branched-chain amino acid metabolism; (2) biosynthesis of phenylalanine, tyrosine, and tryptophan within aromatic amino acid metabolism; (3) protein digestion and absorption; and (4) arginine biosynthesis (Figure 3). These findings indicate the presence of significant intestinal amino acid metabolic disturbances in children with migraine.

### 3.4 Analysis of SCFAs

**Figure 4:**
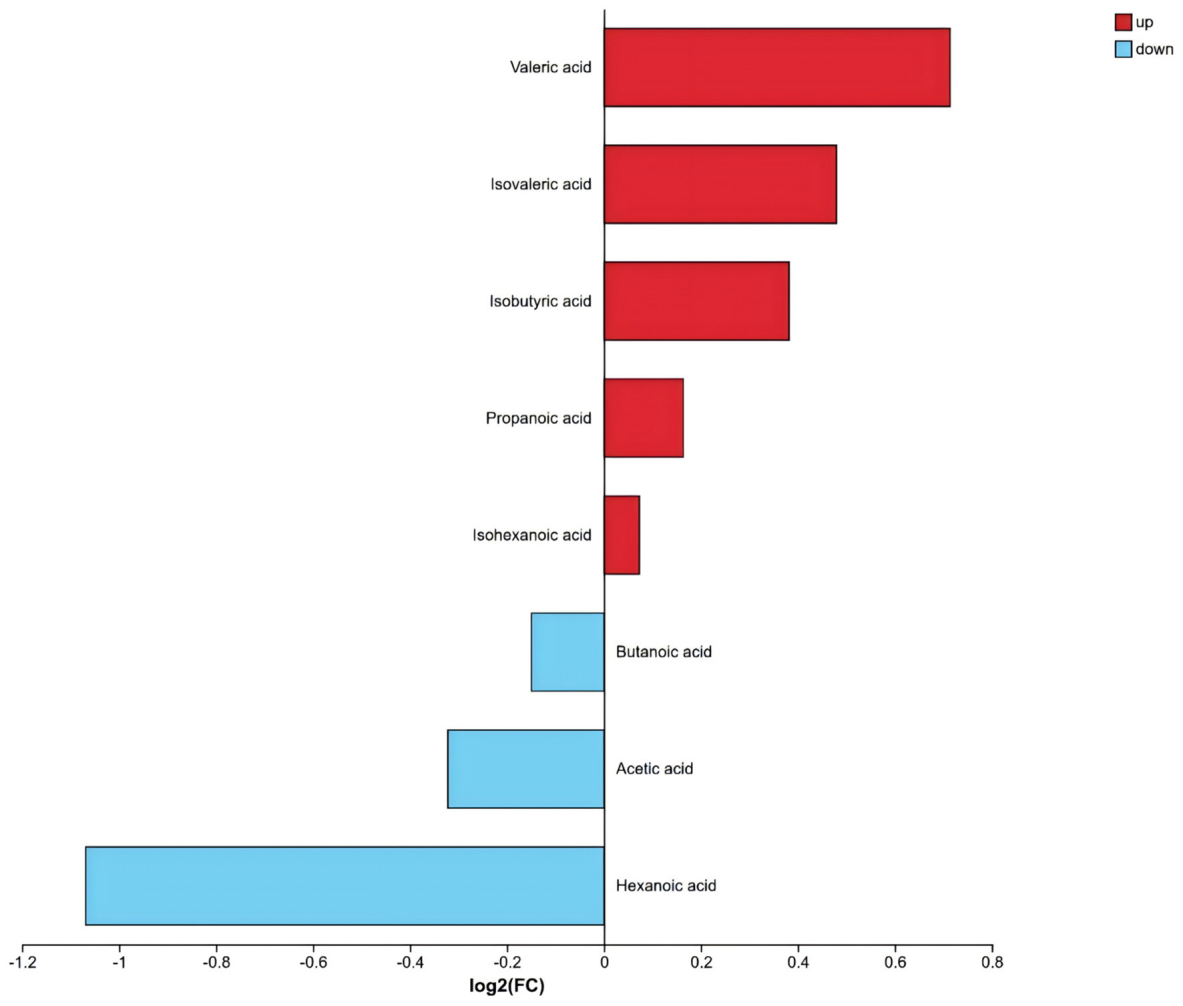
SCFAs-difference multiples histogram. *Note:* The abscissa represents log_2_ (fold change), and the ordinate shows the metabolite names. Red bars indicate up-regulated metabolites, blue bars indicate down-regulated metabolites, and the bar length corresponds to the log_2_ (fold-change) value.

**Figure 5:**
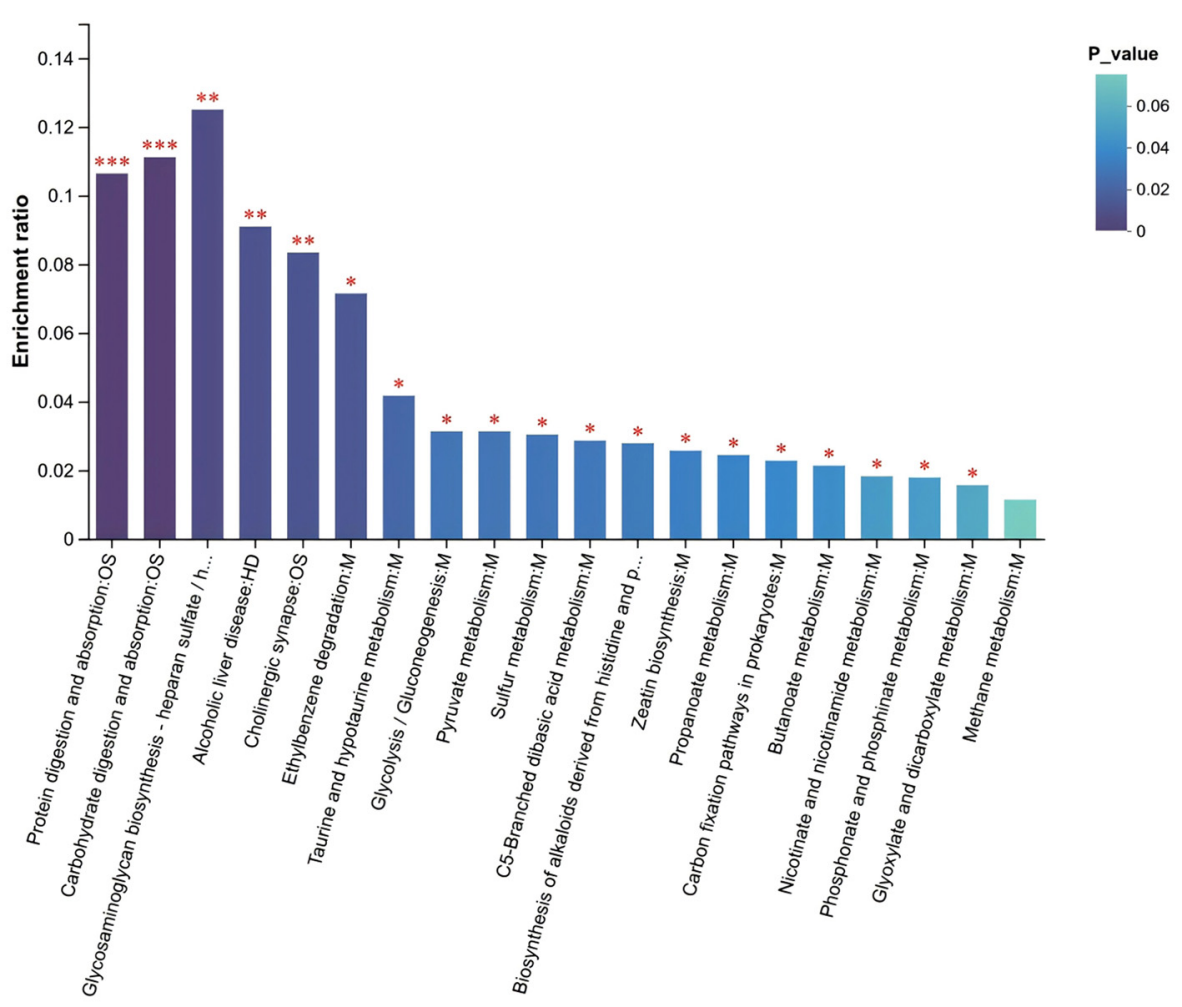
Enrichment analysis of differential SCFAs-related KEGG pathways. *Note:* The abscissa indicates the pathway names, and the ordinate represents the enrichment ratio, defined as the number of metabolites enriched in a given pathway (Metabolite number) divided by the total number of metabolites annotated to that pathway (Background number). A higher ratio indicates a greater degree of enrichment. The color gradient of the bars reflects the statistical significance of enrichment; darker colors denote more significantly enriched KEGG terms, with ***P < 0.001, **P < 0.01, and *P < 0.05.

Targeted absolute quantification of SCFAs (ng/mg) showed limited differences in fecal SCFA profiles between children with migraine and healthy controls. Compared with healthy controls, valeric acid and propanoic acid tended to be higher in the migraine group, whereas acetic acid and butanoic acid tended to be lower (Table 2, Figure 4). Overall, however, the between-group differences were limited, and differences in most individual SCFAs did not reach statistical significance. KEGG enrichment analysis showed that SCFA-related metabolites were enriched in the protein digestion and absorption pathway (FDR *P* < 0.001) and the carbohydrate digestion and absorption pathway (FDR *P* < 0.001). In addition, group differences were also observed in the propanoate metabolism and butanoate metabolism pathways (Figure 5).

**Table 2:** Fecal SCFA concentrations in healthy controls and patients with migraine.

Metabolite	Healthy controls (ng/mg)	Migraine group (ng/mg)	FC (migraine/HC)	*P-value*
Acetic acid	2,744.32 (2,117.06, 3,183.80)	2,174.61 (1,339.08, 3,136.73)	0.80	0.05
Propanoic acid	755.10 (679.38, 1,127.15)	841.84 (607.24, 1,277.70)	1.12	0.82
Isobutyric acid	99.73 (31.04, 160.89)	88.32 (42.40, 172.51)	1.30	0.64
Butanoic acid	766.35 (497.24, 1,194.44)	553.65 (359.33, 1,137.91)	0.90	0.34
Isovaleric acid	73.19 (25.95, 140.56)	71.31 (32.85, 131.45)	1.39	0.71
Valeric acid	46.30 (19.02, 144.56)	102.76 (20.00, 187.12)	1.64	0.22
Isohexanoic acid	1.68 (0.07, 5.51)	0.90 (0.15, 4.91)	1.05	0.81
Hexanoic acid	3.45 (1.97, 5.51)	3.49 (1.90, 6.51)	0.48	0.99

### 3.5 Correlation analysis of differential amino acids and SCFAs

**Figure 6:**
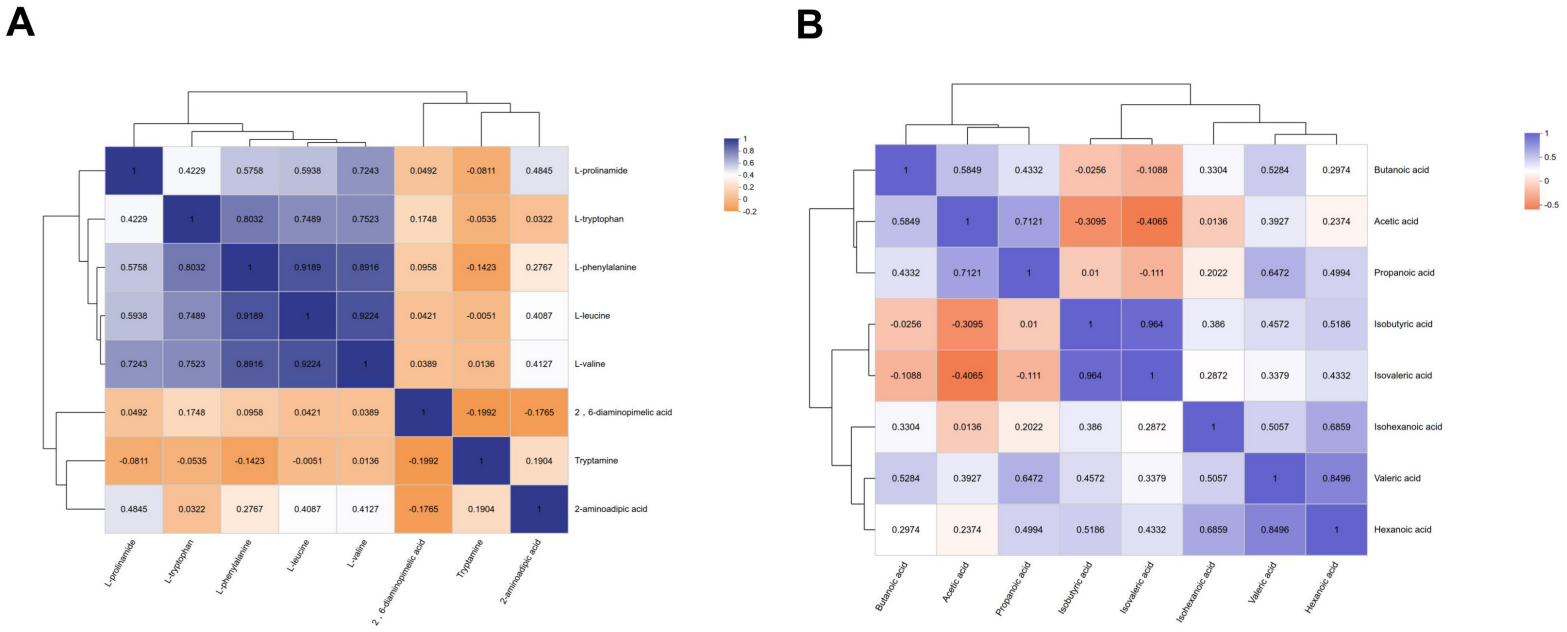
Correlation heatmaps of differential amino acids and short-chain fatty acids in children with migraine. *Note:* **(A)** Heatmap showing the correlations among eight significantly altered amino acids and their derivatives in the migraine group. **(B)** Heatmap showing the correlations among SCFAs in the migraine group.

Spearman correlation analysis was performed on the eight differential metabolites in the migraine group (Figure 6A). The results showed that L-prolinamide, L-tryptophan, L-phenylalanine, L-leucine, and L-valine were overall significantly positively correlated. Among them, L-phenylalanine was strongly positively correlated with L-leucine (*r* = 0.9189, *P* < 0.001) and L-valine (*r* = 0.8916, *P* < 0.001), while the strongest correlation was observed between L-leucine and L-valine (*r* = 0.9224, *P* < 0.001). In addition, L-tryptophan was also significantly positively correlated with L-phenylalanine, L-leucine, and L-valine (*r* = 0.8032, 0.7489, and 0.7523, respectively; all *P* < 0.001), and L-prolinamide likewise showed moderate to strong positive correlations with the above metabolites (*r* = 0.4229–0.7243, *P* < 0.05).

Although between-group differences in individual SCFAs did not reach statistical significance, Spearman correlation analysis revealed different within-group correlation patterns in the two groups. In the healthy control group, isobutyric acid showed a very strong positive correlation with isovaleric acid (*r* = 0.98, *P* < 0.001) and relatively stable correlations with valeric acid and hexanoic acid. In the migraine group, acetic acid was significantly positively correlated with propanoic acid (*r* = 0.71, *P* < 0.001), whereas acetic acid was significantly negatively correlated with isovaleric acid (*r* = −0.41, *P* = 0.026). A negative correlation trend was also observed between acetic acid and isobutyric acid, although this did not reach statistical significance (*r* = −0.31, *P* = 0.0961). In addition, isobutyric acid remained strongly positively correlated with isovaleric acid (*r* = 0.96, *P* < 0.001), and valeric acid was significantly positively correlated with hexanoic acid (*r* = 0.85, *P* < 0.001). Given that most individual SCFA comparisons between groups were not statistically significant, these correlation patterns should be interpreted cautiously and regarded as exploratory observations.

### 3.6 ROC curve analysis of differential metabolites

**Figure 7:**
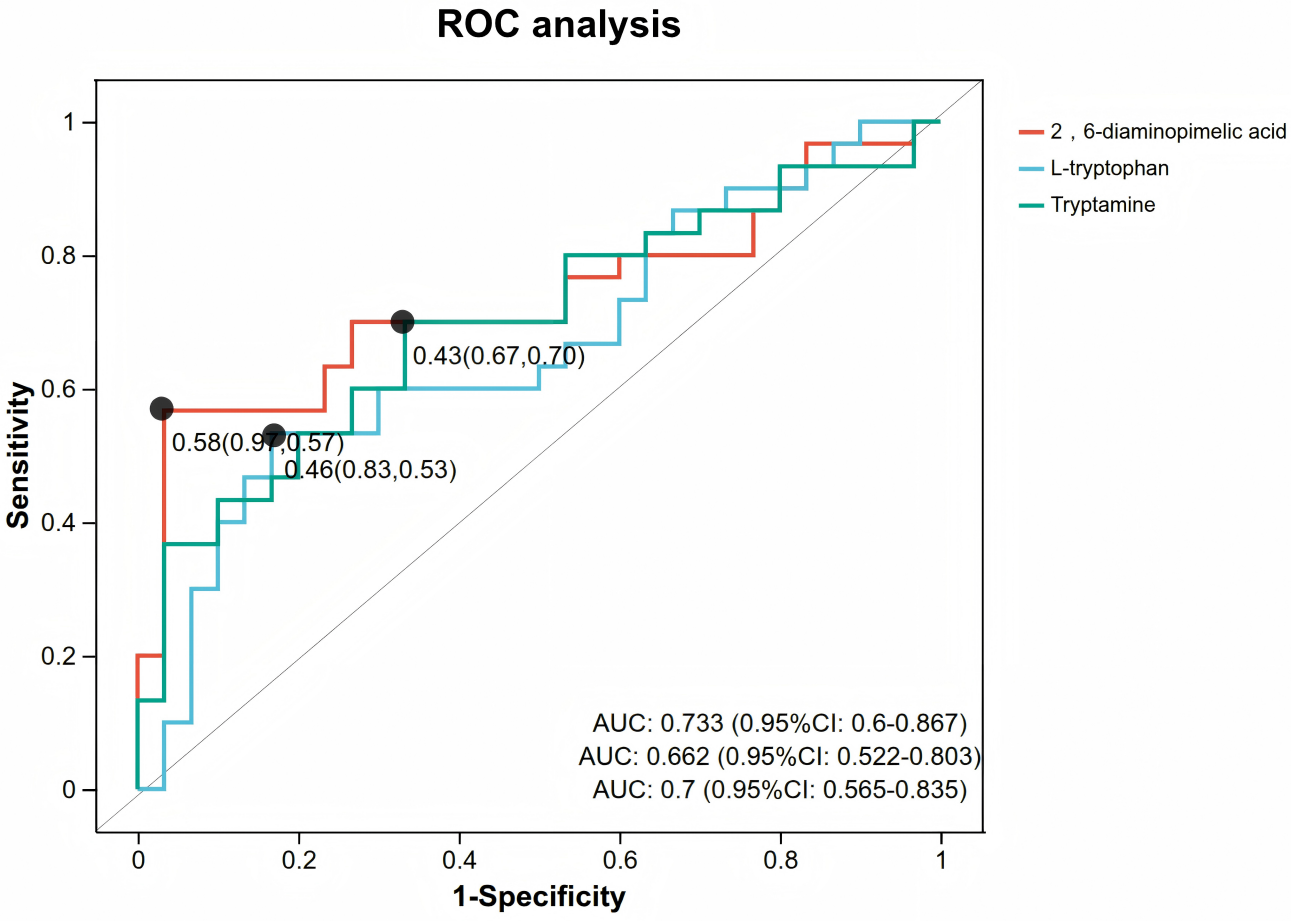
ROC curve analysis of differential metabolites. *Note:* The ROC curves evaluate the diagnostic performance of potential biomarkers for pediatric migraine. The area under the curve (AUC) represents the discriminative ability of the metabolites. An AUC closer to 1 indicates a higher diagnostic value, while an AUC of 0.5 indicates no predictive ability.

ROC analysis showed that tryptamine (AUC = 0.70, 95% CI: 0.56–0.84), 2,6-diaminopimelic acid (AUC = 0.73, 95% CI: 0.60–0.87), and L-tryptophan (AUC = 0.66, 95% CI: 0.52–0.80) had modest discriminative performance in distinguishing children with migraine from healthy controls. Among them, tryptamine and 2,6-diaminopimelic acid showed statistically significant discriminatory ability; however, their AUC values indicate only modest performance. No significant differences were observed in the ROC analysis of SCFAs (Figure 7).

## 4 Discussion

In this study, targeted metabolomics was employed to reveal altered fecal metabolic profiles in children with migraine. We investigated alterations in intestinal metabolites among pediatric migraine patients from the Hebei region of China and observed differences in both amino acid and SCFA metabolic profiles. These findings suggest that pediatric migraine may be associated with altered fecal metabolic patterns.

KEGG pathway enrichment analysis revealed that the eight differentially abundant amino acids were significantly enriched in key metabolic pathways, including “biosynthesis of phenylalanine, tyrosine, and tryptophan” and “biosynthesis and degradation of valine, leucine, and isoleucine.” This result is consistent with previous observations of altered serum levels of valine and leucine in migraine patients reported by (Domitrz et al. 2015). The overall metabolic profile suggests that amino acid-related metabolic alterations may be associated with altered metabolic patterns in children with migraine.

Nitric oxide (NO) is considered a trigger factor for migraine (Olesen, 2008), and the occurrence of cortical spreading depression (CSD) can lead to NO release and neurovascular changes (Busija et al., 2008). In our study, we observed enrichment of the arginine biosynthesis pathway in children with migraine. Arginine serves as a substrate for nitric oxide synthase (Maxwell et al., 2001), participates in the urea cycle *in vivo*, and is closely associated with immune function and antioxidant capacity (Rezaei et al., 2021). Elevated levels of NO can impair mitochondrial respiratory chain function by inhibiting key enzymatic components. Migraine is widely regarded as a condition in which oxidative stress exceeds the body's antioxidant defenses (Fila et al., 2019; Gross et al., 2019). Therefore, we speculate that the enrichment of arginine-related metabolism observed in this study may be linked to NO-related mechanisms previously implicated in migraine. However, this possibility remains hypothetical because NO levels and downstream signaling activity were not directly assessed (Olesen, 2024).

L-Phenylalanine serves as a precursor for the synthesis of dopamine and norepinephrine (Kim et al., 2022; Marchiosi et al., 2020). Tryptophan is involved in serotonin production (Maffei, 2020) and can be readily oxidized to tryptamine. Alterations in tryptophan-, tryptamine-, and phenylalanine-related metabolism may be relevant to neurotransmitter pathways previously implicated in migraine (Andreou and Pereira, 2023; Otaru et al., 2024; Suzuki et al., 2022). Additionally, as a metabolite related to bacterial cell wall components (Czauderna and Kowalczyk, 1999; Pasquina-Lemonche et al., 2020), altered levels of 2,6-diaminopimelic acid may reflect structural changes in the gut environment. Regarding SCFAs, butyric acid and acetic acid are important energy substrates for maintaining intestinal barrier integrity (Minton, 2022; VanHook, 2015); their levels were observed to be reduced. Overall, while some SCFA pathways were enriched, the majority of individual SCFA alterations lacked statistical significance.

In the present study, valeric acid showed an increasing trend in the migraine group, although the between-group difference did not reach statistical significance. More broadly, SCFA-related pathway enrichment analysis indicated associations with protein digestion and absorption, carbohydrate digestion and absorption, as well as propanoate and butanoate metabolism. In addition, correlation analysis further suggested that the intra-group association patterns of SCFAs differed between healthy controls and children with migraine. These findings imply that alterations in SCFAs in pediatric migraine may be better reflected at the level of overall metabolic patterns rather than in isolated changes of individual metabolites. The combined alterations observed in amino acid and SCFA profiles suggest that pediatric migraine may be associated with changes in fecal metabolic patterns.

However, these interpretations should be viewed in light of several notable limitations. First, this was an exploratory cross-sectional study with a relatively small sample size (60 participants) from a single geographic region, which may have limited the ability to detect subtle metabolic variations or perform robust stratified analyses by migraine subtypes. Second, potentially relevant confounding factors, including body mass index (BMI), pubertal stage, dietary intake, gastrointestinal symptoms, recent stress exposure, and possible longer-term antibiotic effects, were not fully controlled. This is a major limitation, as dietary habits directly shape the availability of metabolic substrates (David et al., 2013), while BMI and gastrointestinal symptoms can independently alter intestinal transit times and fecal metabolite excretion (Gacesa et al., 2022). Furthermore, psychological stress is known to influence systemic metabolism and gut motility (Foster et al., 2017). The observed metabolic variations could partially reflect these clinical and environmental confounders. Third, although some metabolites showed modest discriminative performance in ROC analysis, these findings were derived from a single cohort and were not externally validated. Finally, because microbiome compositional data were not available, the observed fecal metabolic alterations cannot be directly linked to specific microbial taxa. Future larger multicenter studies integrating longitudinal sampling and microbiome-metabolome analyses are needed to further validate these findings.

## 5 Conclusions

In conclusion, this study found that children with migraine exhibited altered fecal metabolic profiles, mainly characterized by changes in specific amino acid-related metabolites. Specifically, increased metabolic levels of phenylalanine, tryptophan, valine, leucine, and 2,6-diaminopimelic acid were observed. Regarding SCFAs, although most individual metabolites lacked statistical significance, subtle alterations were noted at the broader pathway and intra-group correlation levels. Overall, these findings suggest that pediatric migraine may be associated with distinct metabolite-level patterns in the gut. Future multicenter studies incorporating larger sample sizes, strict control of clinical confounders, and multi-omics designs are required to validate these observational findings and explore their biological significance.

## Data Availability

The original contributions presented in the study are included in the article/Supplementary material, further inquiries can be directed to the corresponding author.
