# Supplementary material for: Fecal amino acid and short-chain fatty acid profiles in children with migraine: a targeted metabolomics study
Source: Front Microbiol. 2026 May 8;17:1788386. doi: 10.3389/fmicb.2026.1788386 (PMC13195267; doi:10.3389/fmicb.2026.1788386)
Supplement: Supplementary file 1 [file Supplementary_File_1.docx]

Supplementary Material

# Detection of amino acids and their derivatives.

Initially, a total of 68 amino acids and their derivatives were targeted for qualitative and quantitative analysis. A standard stock solution for each of the 68 amino acids and their derivatives was prepared by accurately weighing the corresponding reference standard and dissolving it in either water or methanol. An appropriate volume from each stock solution was then combined to prepare a mixed standard, which was subsequently diluted with 50% acetonitrile in water to the appropriate concentrations, yielding the working standard solutions.

Sample pretreatment and extraction were performed as follows: approximately 10 mg of each sample (based on the actual sampled amount) was accurately weighed. A steel ball and 1000 μL of 90% methanol were added, and the sample was ground for 6 min in a refrigerated freeze grinder maintained at −10 °C (50 Hz) to ensure a stable low-temperature environment and prevent metabolite degradation. The homogenate was centrifuged at 14,000 rcf at 4 °C for 20 min. A 10 μL aliquot of the supernatant was transferred to a 1.5 mL centrifuge tube and dried under a gentle stream of nitrogen.

For the derivatization process, the residue was reconstituted in 50 μL of a 50% acetonitrile aqueous solution and vortexed for 1 min. Subsequently, 30 μL of a 10 mg/mL dansyl chloride solution and 40 μL of a 0.5 M sodium carbonate-ammonium bicarbonate buffer (pH 9.0) were added. The tube was sealed, vortexed vigorously for 1 min, and incubated at 60 °C for 30 min in a thermostatic oscillator. After incubation, the mixture was left at room temperature for 5 min. Then, 10 μL of a 0.25 M sodium hydroxide solution was added, followed by a second incubation at 60 °C for 10 min to remove excess dansyl chloride. After cooling to room temperature, 70 μL of a 10% formic acid solution was added to stabilize the derivatives, and the mixture was vortexed and centrifuged at 14,000 rcf at 4 °C for 15 min. The resulting supernatant was finally collected into an injection vial for LC-MS/MS detection.

For the derivatization process, the residue was reconstituted in 50 μL of a 50% acetonitrile aqueous solution and vortexed for 1 min. Subsequently, 30 μL of a 10 mg/mL dansyl chloride solution and 40 μL of a 0.5 M sodium carbonate-ammonium bicarbonate buffer (pH 9.0) were added. The tube was sealed, vortexed vigorously for 1 min, and incubated at 60 °C for 30 min in a thermostatic oscillator. After incubation, the mixture was allowed to stand at room temperature for 5 min. To remove excess dansyl chloride, 10 μL of a 0.25 M sodium hydroxide solution was added, followed by vortexing and a second incubation at 60 °C for 10 min. After the mixture cooled to room temperature, 70 μL of a 10% formic acid solution was added to neutralize the reaction and stabilize the derivatives. The mixture was then vortexed and centrifuged at 14,000 rcf and 4 °C for 15 min. The resulting supernatant was collected into an injection vial for detection.

Qualitative and quantitative detection of the target analytes was performed using an UHPLC-Qtrap system. The analytical platform consisted of an ExionLC AD liquid chromatography system coupled with a SCIEX QTRAP 6500+ mass spectrometer. Chromatographic separation was achieved using a Waters HSS T3 column (2.1 × 100 mm, 1.8 μm) maintained at 40 °C. The injection volume was 2 μL. Mobile phase A consisted of 0.1% formic acid in water, and mobile phase B consisted of 0.1% formic acid in methanol. Mass spectrometry was operated in positive electrospray ionization (ESI+) mode. The ion source parameters were optimized as follows: Curtain Gas (CUR): 35 psi; Collision Gas (CAD): Medium; IonSpray Voltage (IS): +5000 V; Temperature (TEM): 350 °C; Ion Source Gas 1 (GS1): 60 psi; Ion Source Gas 2 (GS2): 60 psi.

# Short-Chain Fatty Acid Detection

Initially, a total of 8 short-chain fatty acids (SCFAs)—including acetic, propionic, butyric, isobutyric, valeric, isovaleric, hexanoic, and isohexanoic acid—were targeted for qualitative and quantitative analysis. Preparation of mixed standards: Acetic acid, propionic acid, butyric acid, isobutyric acid, valeric acid, and isovaleric acid (10 mg each), along with hexanoic acid and isohexanoic acid (1 mg each), were accurately weighed. Each was then dissolved in and brought to a final volume of 1 mL with a 50% acetonitrile aqueous solution, followed by vortex mixing to obtain individual standard stock solutions. An appropriate volume from each single standard solution was combined and diluted to 1 mL to prepare mixed standard stock solution A. Mixed standard stock solution B was subsequently obtained by performing a 50-fold dilution of mixed standard stock solution A.

Standard curve pretreatment: To 40 µL of mixed standard stock solution B, 20 µL of 200 mM 3-nitrophenylhydrazine hydrochloride (3NPH·HCl) and 20 µL of 120 mM 1-ethyl-3-(3-dimethylaminopropyl)carbodiimide hydrochloride (EDC·HCl) solution (containing 6% pyridine) were added. Both derivatization reagents were dissolved in a 50% acetonitrile aqueous solution. The reaction was conducted at 40 °C for 30 min. The mixture was then diluted to a final volume of 200 µL with the 50% acetonitrile aqueous solution and subsequently subjected to serial dilution to prepare the working standard solutions (L1-L14) in 1.5 mL EP tubes.

Sample pretreatment: Approximately 20 mg of each sample (based on the actual sampled weight) was accurately weighed. Then, 500 µL of extraction solvent (methanol: water = 4:1, v/v) was added, and the sample was ground for 6 min using a freeze grinder (−10 °C, 50 Hz) to ensure a stable low-temperature environment and prevent metabolite degradation, followed by low-temperature ultrasonication for 30 min (5 °C, 40 kHz). The homogenate was allowed to stand at −20 °C for 30 min to further facilitate protein precipitation and then centrifuged at 13,000 rcf and 4 °C for 15 min. A 20 µL aliquot of the supernatant was mixed with 20 µL of 200 mM 3NPH·HCl and 20 µL of 120 mM EDC·HCl (containing 6% pyridine) solution. Derivatization was carried out at 40 °C for 30 min. The reaction mixture was finally diluted to 1000 µL with a 50% acetonitrile aqueous solution prior to detection.

The specific analytical parameters for short-chain fatty acids were as follows: An ExionLC AD system was used with a Waters BEH C18 column (150 × 2.1 mm, 1.7 µm). The column temperature was maintained at 40 °C, and the injection volume was 2 µL. The mobile phases consisted of 0.1% formic acid in water (mobile phase A) and 0.1% formic acid in acetonitrile (mobile phase B). Detection was performed in negative electrospray ionization (ESI−) mode using a SCIEX QTRAP 6500+ mass spectrometer. The ion source parameters were set as follows: Curtain Gas (CUR): 35, Collision Gas (CAD): Medium, IonSpray Voltage (IS): −4500 V, Temperature (TEM): 450 °C, Ion Source Gas 1 (GS1): 40, Ion Source Gas 2 (GS2): 40.

Table 1.instruments

| Name of instrument | Company |
| --- | --- |
| QTRAP® 6500+ triple quadrupole mass spectrometer  ExionLC AD system  Waters HSS T3(2.1x150 mm,1.8μm) liquid chromatography column  Wonbio-96E Freeze Grinding Machine  SBL-10DT ultrasonic cleaning machine 300W-10L  Centrifuge 5430R high-speed freezing centrifuge  NewClassic MFMS105DU electronic balance  DW-86L390 type cryopreservation refrigerator  THZ-D Thermostatic Oscillator | SCIEX Company  SCIEX Company  American Waters Inc.  Shanghai Wanbai Biotechnology Co., Ltd.  Ningbo Xinzhi Biotechnology Co., Ltd.  Eppendorf, Germany ;  Swiss company METTLER  Aucma Corporation Limited  Suzhou Peiying Experimental Equipment Co., Ltd. |

Table 2. Reagents

| Reagent name | Company |
| --- | --- |
| Acetonitrile (chromatographic grade) | Fisher Company |
| Methanol (chromatographic grade) | Fisher Company |
| Water (chromatographic grade)  Formic acid  Sodium carbonate  Sodium bicarbonate  Dansyl chloride  Sodium hydroxide | Fisher Company  CNW Company  Adamas Company  Adamas Company  Sigma Company  Guoyao Group Chemical Reagent Co., Ltd. |
